# Synthesis and *in Vitro* Antiproliferative Activity of New Phenylaminoisoquinolinequinones against Cancer Cell Lines

**DOI:** 10.3390/molecules18010721

**Published:** 2013-01-08

**Authors:** Virginia Delgado, Andrea Ibacache, Verónica Arancibia, Cristina Theoduloz, Jaime A. Valderrama

**Affiliations:** 1 Facultad de Química, Pontificia Universidad Católica de Chile, Casilla 306, Santiago 26094411, Chile; 2 Facultad de Ciencias de la Salud, Universidad de Talca, Talca 3460000, Chile; 3 Instituto de Etno-Farmacología (IDE), Universidad Arturo Prat, Casilla 121, Iquique 1100000, Chile

**Keywords:** phenylaminoisoquinoline-5,8-quinones, regioselectivity, half-wave potential, antiproliferative activity, SAR analysis

## Abstract

A variety of phenylaminoisoquinolinequinones were synthesized and tested for their antiproliferative activity against three human-tumor derived cancer cell lines. The new aminoquinones were prepared from 4-methoxycarbonyl-3-methylisoquinoline-5,8-quinone (**1**) via acid-induced amination and bromination reactions. Remarkable differences in antiproliferative activity were observed depending upon the location and donor capacity of the substituted phenylamino group at the quinone nucleus. The effect of the substituents on the biological activity is discussed in terms of the donor-acceptor interactions which were evaluated through the redox properties of the aminoquinones.

## 1. Introduction

The molecular framework of a variety of naturally occurring antibiotics contains an aminoquinoline- and aminoisoquinoline-5,8-quinone moiety as the key structural component (*i.e.*, streptonigrin [1,2], lavendamycin [3,4], cribrostratin 3 [5], caulibugulones A–C [6] and mansouramycins A–C [7]). This structural array has stimulated the synthesis of novel aminoquinoline- and aminoisoquinoline-5,8-quinones [8,9,10,11,12]. The main target of these synthetic efforts is to extend the spectrum of antiproliferative activity on cancer cells. In the light of these facts we and other authors have reported the preparation and antiproliferative evaluation of 6,7-substituted aminoquinoline- and aminoisoquinolinequinones. Evidences arising from these studies demonstrate that insertion and change of location of alkylamino and halogen substituents in the quinone nucleus of the corresponding *N*-heterocyclic cores induce difference in the antiproliferative activity [13,14,15].

In the search for new aminoquinones endowed with high antiproliferative potency we are interested in the evaluation of the effect of the insertion and change of location of *p*-substituted phenylamino and bromine groups at the 6- and 6,7-positions of an isoquinoline-5,8-quinone chemotype on the antiproliferative activity. In this paper, we present the synthesis of a series of 6,7-substituted isoquinolinequinones, which were evaluated in terms of their *in vitro* antiproliferative properties against a panel of three cancer cell lines.

## 2. Results and Discussion

### 2.1. Chemistry

**Scheme 1:**
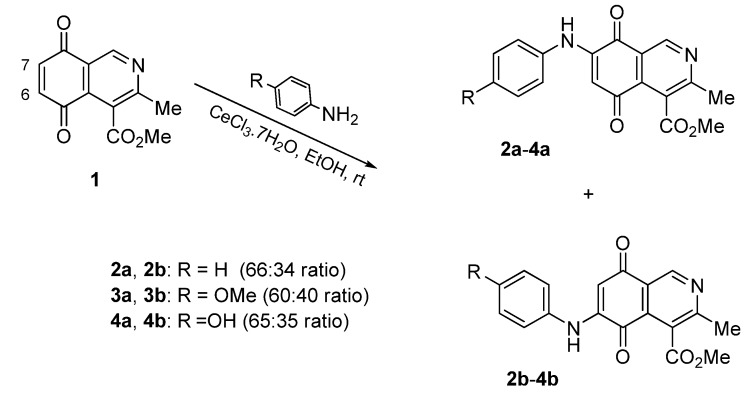
Synthesis of 6- and 7-phenylaminoisoquinolinequinones **2a**,**b**–**4a**,**b**.

Isoquinolinequinone **1** was selected as precursor of the designed phenylaminoisoquinolinequinones taking into account its reactivity with alkylamines, which provides simultaneous access to 6- and 7-aminoisoquinolinequinones [12]. The synthesis of **1** was accomplished in 86% yield from commercially available 2,5-dihydroxybenzaldehyde and methyl aminocrotonate, through a one-pot procedure previously developed in our laboratory [12] (Scheme 1). The preparation of compounds **2a**,**b** was initially explored by reaction of quinone **1** with aniline in ethanol at room temperature. The reaction went to completion in 3.5 h giving a 95:5 mixture of the expected regioisomers **2a**,**b**, albeit in 37% yield. The ratio between regioisomers **2a**,**b** was evaluated by ^1^H-NMR using the signals of the protons at C-1 of each regioisomer, located at 9.24 and 9.29 ppm, respectively. A better result in the preparation of regioisomers **2a**,**b** was obtained when the amination reaction of **1** was carried out in ethanol in the presence of 5 mmol % of CeCl_3_·7H_2_O. Under these conditions, the reaction was clean and fast (2 h) to give a 66:34 mixture of isomers **2a**,**b** in 74% yield. Pure samples of compounds **2a**,**b** were achieved by column chromatography. These results clearly demonstrate that the catalyst improves the yield of the amination reaction and changes the regioselectivity, favoring the formation of the minor regioisomer **2b**.

Based on the above Lewis acid-induced procedure, phenylaminoisoquinolinequinones **3a**,**b** and **4a**,**b** were prepared by amination reaction of quinone **1** with *p*-anisidine and *p*-aminophenol, respectively. In these experiments, pure samples of the corresponding regioisomers **3a**,**b** and **4a**,**b** were isolated by column chromatography.

The structures of the new compounds were established on the basis of their nuclear magnetic resonance (^1^H-NMR, ^13^C-NMR, 2D-NMR) and high resolution mass spectra (HRMS). The position of the nitrogen substituent in these aminoquinones was determined by means of HMBC experiments. The location of the nitrogen group at C-7 in compounds **2a**, **3a** and **4a** was deduced by the ^3^*J*_C_,_H_ couplings between the carbon at C-8 with the protons at C-1, at C-6 and that of the NH group. In the case of aminoquinones **2b**, **3b** and **4b** the location of the nitrogen substituent at C-6 was established by ^3^*J*_C,H_ coupling between the carbon at C-5 with the proton at C-7 and the proton of the NH group [12,16].

**Scheme 2:**
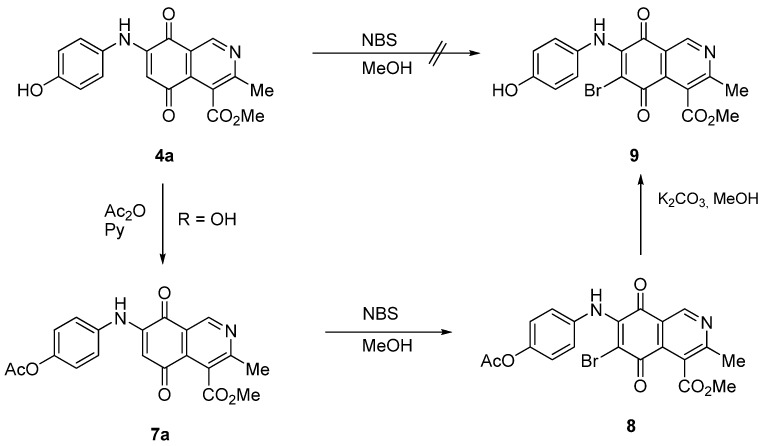
Synthesis of the bromine derivative **9** from phenylaminoquinone **4a**.

The insertion of a bromine atom in phenylaminoisoquinolinequinones **2**–**4** was attempted by reaction of aminoquinones **2**–**4** with *N*-bromosuccinimide (NBS). The substitution reaction of aminoquinones **2a**,**b** and **3a**,**b** proceeded under mild conditions to give the expected bromoquinones **5a**,**b** and **6a**,**b** (Table 1). Regarding the bromination attempts of arylaminoquinone **4a**, by TLC was observed the disappearance of **4a** with concomitant appearance of a yellow spot. However, after the aqueous work-up of the mixture reaction, aminoquinone **4a** was recovered and no reaction products were detected (TLC, ^1^H-NMR). A plausible explanation for the lack of bromination of compound **4a** could be attributed to the oxidation of the *p*-hydroxyphenylamino group of **4a** with NBS [13,14,15] to give the corresponding electron-attracting iminoquinoyl group, which probably inhibits the bromination reaction at the quinone double bond and, under aqueous media, undergoes a reversible reduction to the hydroxyphenylamino group. In order to avoid the interference of the phenylamino substituent on the bromination reaction at the 6-position of **4a**, we attempted to prepare the target brominated regioisomer **9** through a three step sequence, which involved the protection of the hydroxyl group in **4a**, as the acetate, followed by bromination of **7a** with NBS and further base-induced deprotection of acetyl group in **8**, of the hydroxyl group (Scheme 2). This strategy was successful and the expected bromine derivative **9** was isolated in 33% total yield (Table 1).

**Table 1:** Preparation of phenylaminobromine derivatives **5a**,**b**, **6a**,**b**, **8** and **9**.

Substrate	Bromine derivative N° (Yield %) ^a^	Substrate	Bromine derivative N° (Yield) ^a^
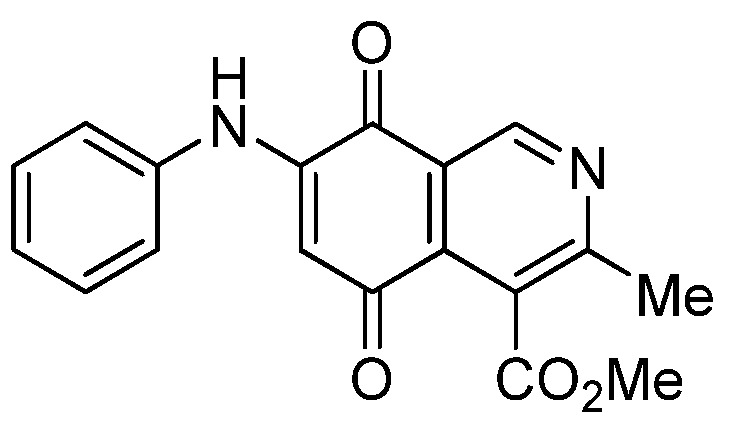	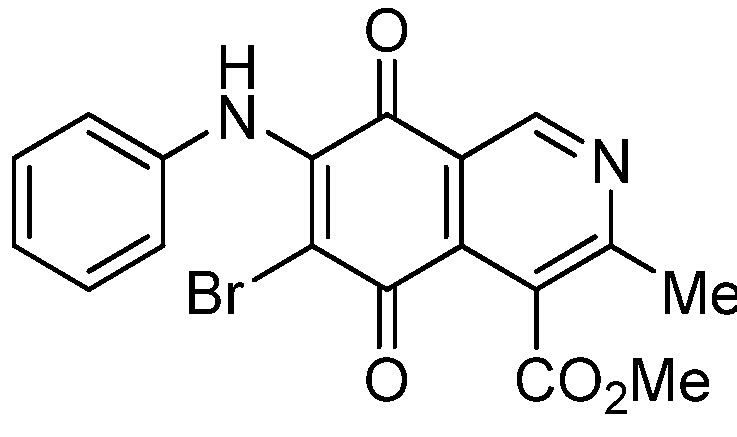	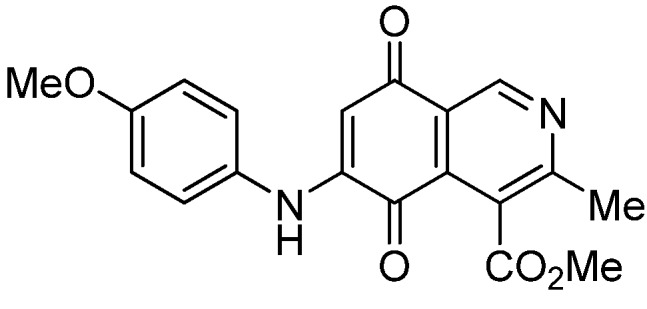	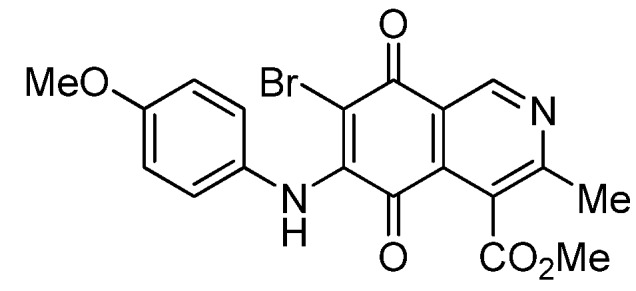
2a	5a (87)	3b	6b (78)
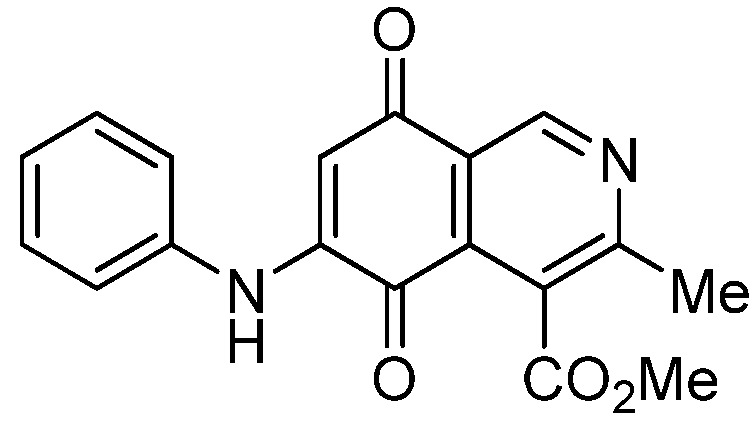	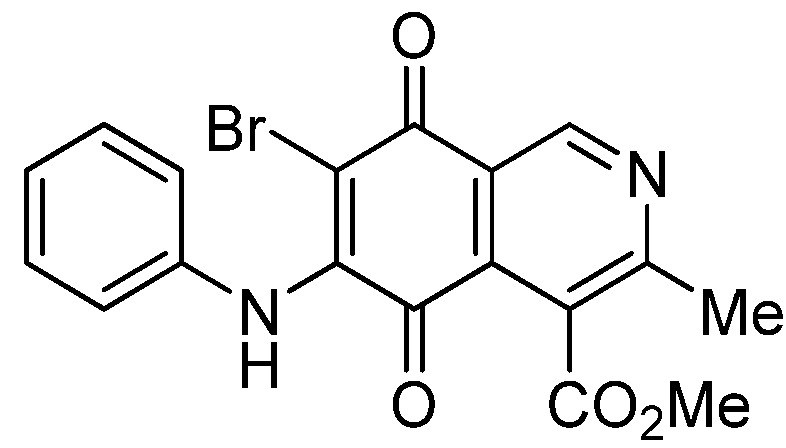	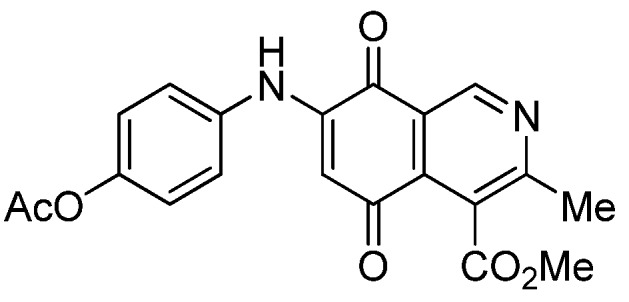	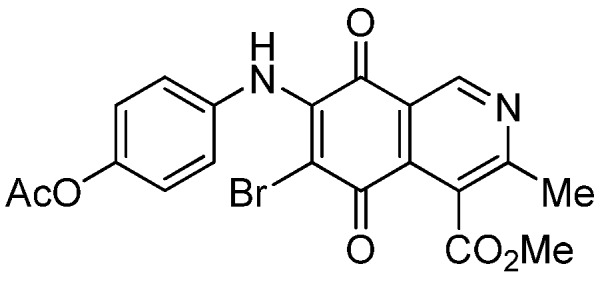
2b	5b (74)	7a	8 (56)
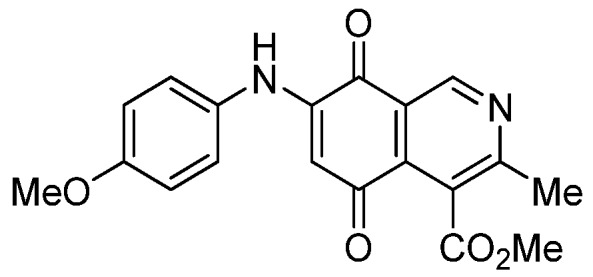	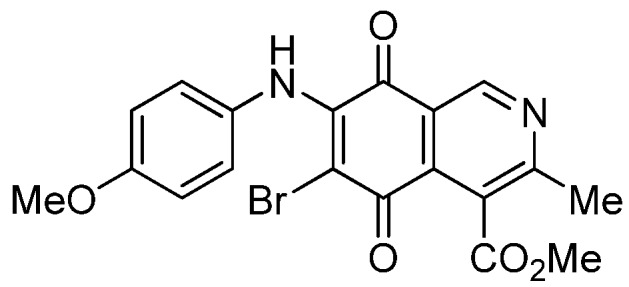	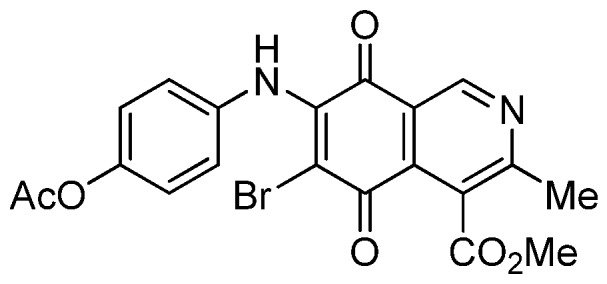	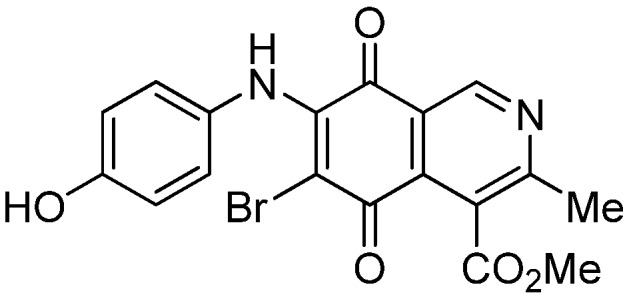
3a	6a (80)	8	9 (64)

^a^ Isolated by column chromatography.

### 2.2. Electrochemical Results

The redox potentials of the new compounds were measured by cyclic voltammetry in acetonitrile as solvent, at room temperature, using a platinum electrode and 0.1 M tetraethylammonium tetrafluoroborate as the supporting electrolyte [16]. Well-defined quasi-reversible waves were observed for the compounds, the cathodic peak related to the reduction of quinone, and the anodic one due to its reoxidation. The voltammograms were run in the potential range 0.0–2.0 V *versus* non-aqueous Ag/Ag^+^. The first half-wave potential values, E^I^_1/2_, evaluated from the voltammograms obtained at a sweep rate of 100 mVs^−1^, are summarized in Table 2.

**Table 2:** Half wave potentials and quinone-proton chemical shifts of **1** and the new compounds.

Compound N°	−E^I^_1/2_ (mV)	−E^II^_1/2_ (mV)	logP ^a^	6 or 7-H ^b^
1	352	1140	0.10	7.04
2a	563	874	0.74	6.39
2b	494	1035	0.74	6.38
3a	551	1083	0.61	6.21
3b	482	1040	0.61	6.20
4a	555	869	0.35	6.20
4b	525	881	0.35	6.18
5a	373	932	1.05	-
5b	518	906	1.05	-
6a	426	795	0.92	-
6b	373	953	0.92	-
7a	521	1058	0.32	6.34
7b	544	711	0.32	6.34
8	386	944	0.63	-
9	431	777	0.66	-

^a^ Determined by the ChemBioDraw Ultra 11.0 software; ^b^ Recorded in CDCl_3_.

The E^I^_1/2_ values for the first electron, which is related to the formation of the semiquinone radical anion, are in the potential range −563 to −352 mV. The data of Table 2 indicate that the presence of phenylamino substituents at the quinone ring in precursor **1** induces the displacement of the half wave potential of **1** from −352 mV to an upper potential zone located in the range: −563 to −373 mV. The position of the potential values within this range evidently depends on the nature and location of the nitrogen substituents.

Comparison of the first half wave potentials between the pair of regioisomers **2**, **3**, **4** and **7**, reveals that the reduction for the 7-substituted isomers, as **2a**, **3a** and **4a**, occurs at more negative E^I^_1/2_ potentials than that of the corresponding 6-substituted isomers, except for the pair **7**. According to reported precedents on the electronic effect of substituents in 1,4-naphthoquinones on E^I^_1/2_ potentials [17], it can be deduced that for each pair of regioisomers, that containing the nitrogen substituent at 7-position has a greater electron-donor capacity. It is interesting to note that comparison of the chemical shifts of the quinone-proton of compounds **2a**,**b**, **3a**,**b**, and **4a**,**4b** (Table 2) indicate that a nitrogen substituent at the 7-position induces a large shielding effect (lower chemical shift values) on the proton at C-6 compared to the effect of the nitrogen substituent at the 6-position on the proton at C-7. This effect is consistent with the differences in half-wave potentials among the analog pairs.

### 2.3. *In Vitro* Antiproliferative Activity of Phenylaminoisoquinolinequinones again Cancer Cell Lines

Isoquinolinequinones **1**, **2a**,**b**, **3a**,**b**, **4a**,**b**, **5a**,**b**, **6a**,**b**, **7a**,**b** (prepared by acetylation of **4b**), **8** and **9** were evaluated for their *in vitro* antiproliferative activity on a panel of three human cancer cell lines: AGS (gastric), SK-MES-1 (lung), and J82 (bladder) using the conventional microculture tetrazolium reduction assay [18,19,20]. The broad variety of the synthesized compounds was designed in order to gain insight into the influence of nitrogen and halogen groups at the quinone nucleus of the isoquinolinequinone chromophore on the biological activity. Table 3 summarizes the data from these evaluations.

According to the IC_50_ values collected in Table 3, it is evident that the insertion of nitrogen substituents at the quinone double bond of compound **1**, as in **2a**,**b**, **3a**,**b**, **4a**,**b**, and **7a**,**7b** induces a remarkable increase of the antiproliferative activity in all the evaluated cell lines, compared to those of precursor **1**, reaching, in some cases, submicromolar IC_50_ values on the tested cancer cell lines. Among the members of this series, compounds **3b**, **4b**, **7b** and **5a**, exhibit higher antiproliferative activities than etoposide, used as the reference drug in the screening.

The initial structure-activity relationship (SAR) of the series was focused on the nature and location of the phenylamino group at the quinone nucleus of the isoquinolinequinone pharmacophore. The data of Table 3 for compounds **2a**,**b**, **3a**,**b**, **4a**,**b**, and **7a**,**7b**reveal that the nature of the substituent at the *para*-position in the phenylamino group (H, OMe, OH, OAc) has a significant influence on the antiproliferative activity on the tested tumor cell lines. Concerning the effect of the location of the phenylamino groups on the biological activity, it is noteworthy that in the 6-regioisomers **2b**, **3b**, **4b**, and **7b**, the nitrogen substituent exerts a greater effect on the antiproliferative activity of the pharmaco-phore than the corresponding 7-regioisomers **2a**, **3a**, **4a**, and **7a** on the gastric and lung cancer cell lines. This biological effect is opposite on bladder tumor cell line for the pair **2a**,**b** and **7a**,**b** where it was observed that the 7-regioisomers were more potent than the respective 6-regioisomers.

**Table 3:** Antiproliferative activity and half-wave potentials of **1** and their 6,7**-**substituted derivatives.

		IC_50_ ± SEM ^a^ (μM)			
N°	Structure	AGS ^b^	SK-MES1 ^c^	J82 ^d^	−E^I^_1/2_ (eV)
**1**	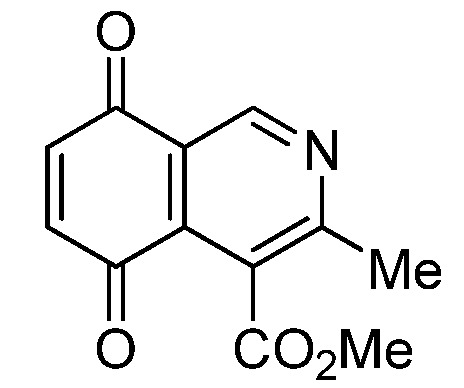	17.34 ± 1.64	25.90 ± 1.60	14.81 ± 0.74	352
**2a**	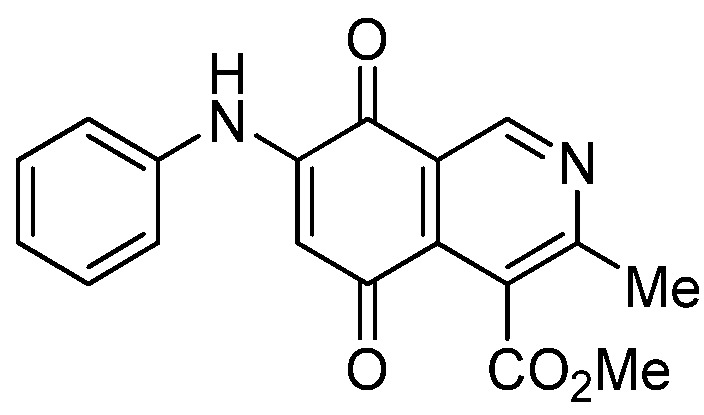	1.10 ± 0.03	4.40 ± 0.09	1.50 ± 0.70	563
**2b**	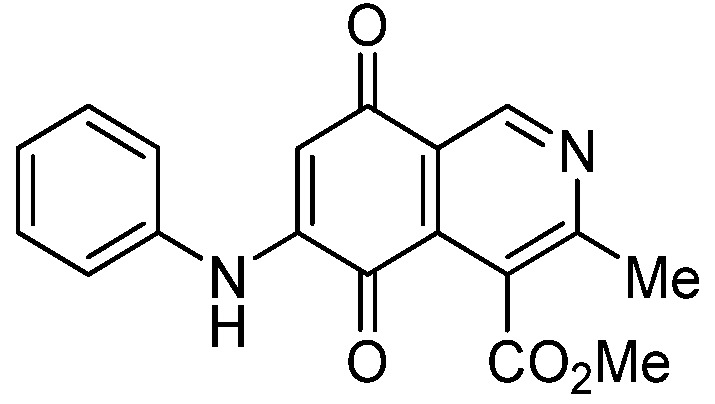	0.57 ± 0.20	2.60 ± 0.30	1.60 ± 0.70	494
**3a**	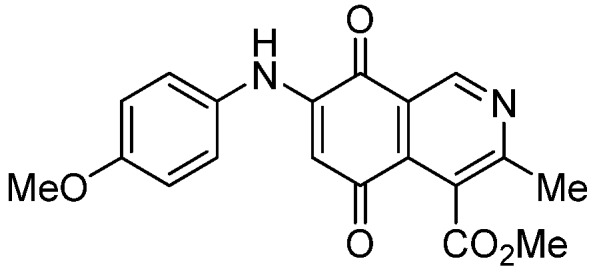	1.10 ± 0.10	3.40 ± 0.60	3.80 ± 0.70	551
**3b**	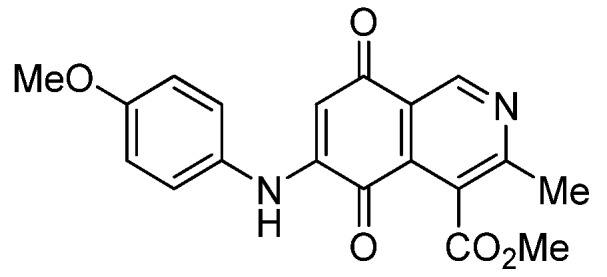	0.32 ± 0.04	1.10 ± 0.09	1.90 ± 0.2 0	482
**4a**	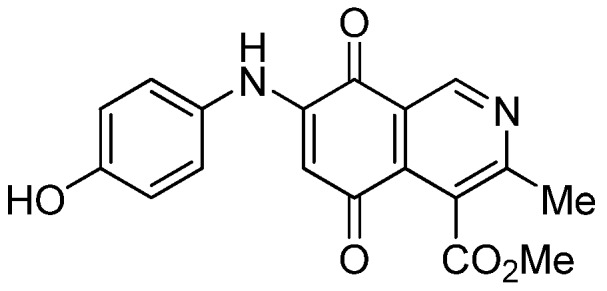	0.72 ± 0.19	2.40 ± 0.12	2.06 ± 0.10	555
**4b**	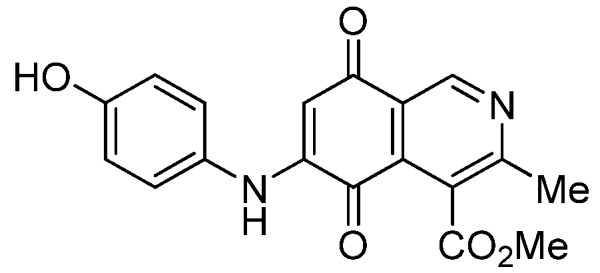	0.24 ± 0.01	0.89 ± 0.05	0.76 ± 0.04	525
**7a**	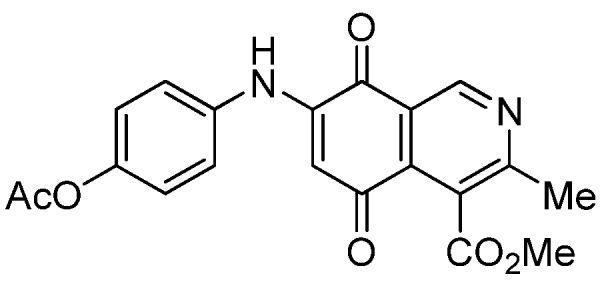	1.01 ± 0.04	0.98 ± 0.05	0.72 ± 0.19	521
**7b**	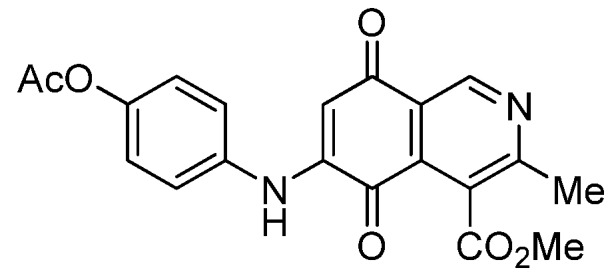	0.29 ± 0.01	0.53 ± 0.04	1.34 ± 0.05	544
**5a**	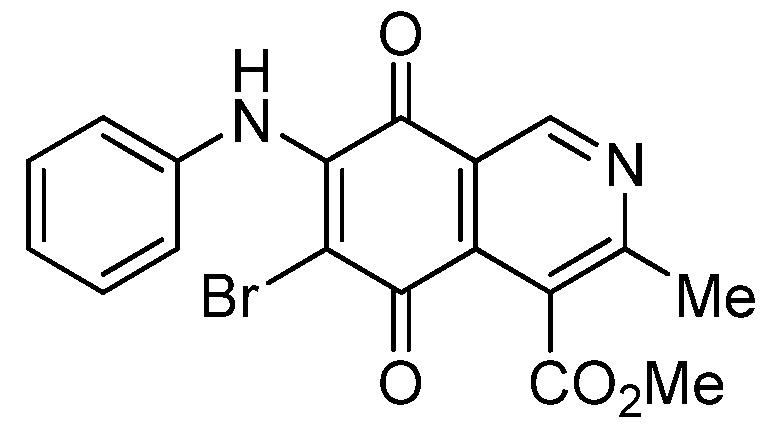	0.70 ± 0.04	0.31 ± 0.01	2.30 ± 0.10	373
**5b**	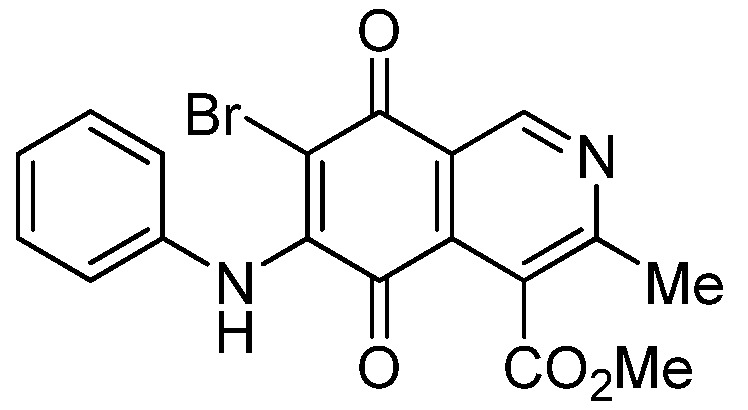	1.84 ± 0.14	4.72 ± 3.23	4.49 ± 0.31	518
**6a**	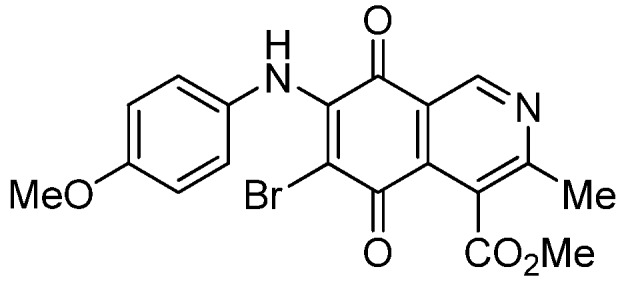	1.11 ± 0.06	1.07 ± 0.04	9.72 ± 0.58	426
**6b**	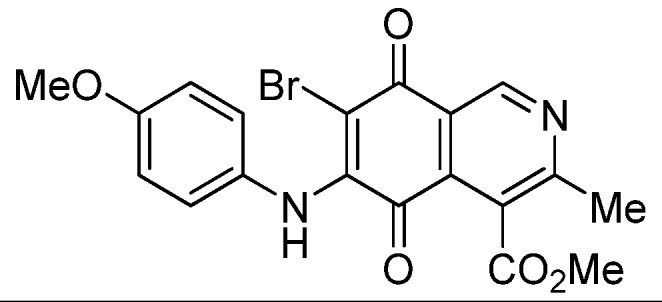	1.80 ± 0.09	1.79 ± 0.11	1.98 ± 0.13	373
**8**	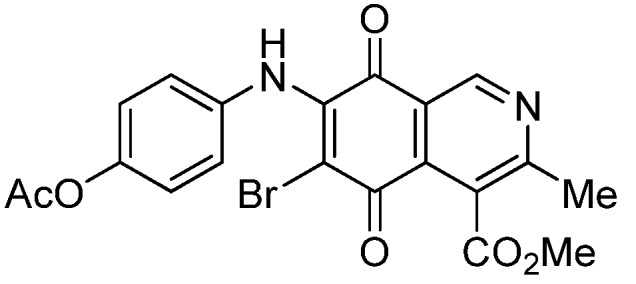	1.73 ± 0.12	3.91 ± 0.27	3.34 ± 0.23	386
**9**	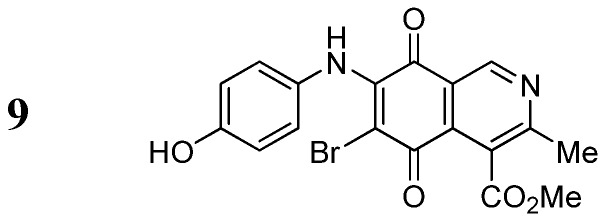	1.10 ± 0.06	2.87 ± 0.14	2.35 ± 0.19	431
-	etoposide	0.58 ± 0.02	1.83 ± 0.09	3.49 ± 0.16	-

^a^ Data represent mean average values for six independent determinations; ^b^ Human gastric adenocarcinoma cell line; ^c^ Human lung cancer cell line; ^d^ Human bladder carcinoma cell line.

Next, the SAR analysis was focused on the effects of insertion of a bromine atom at the quinone ring in the aminoquinones **2a**,**b**, **3a**,**b**, **4a** and **7a**. Comparison of the IC_50_ values of bromine derivatives **5a**,**b**, **6a**,**b**, **8** and **9** with those of their corresponding precursors indicates that the insertion decreases the biological activity with respect to their precursors, except in the case of compound **5a**, which showed a weak increase respect to **2a** in the activity on gastric and lung cancer cell lines.

## 3. Experimental

### 3.1. General

All reagents were commercially available reagent grade and were used without further purification. Melting points were determined on a Stuart Scientific SMP3 apparatus and are uncorrected. ^1^H-NMR spectra were recorded on Bruker AM-200 and Avance-400 instruments in deuterochloroform (CDCl_3_). ^13^C-NMR spectra were obtained in CDCl_3_ at 50 and 100 MHz. Bidimensional NMR techniques and DEPT were used for signal assignment. Chemical shifts are expressed in ppm downfield relative to tetramethylsilane and the coupling constants (*J*) are reported in Hertz. The HRMS spectra were obtained on a Thermo Finnigan spectrometer, model MAT 95XP. Silica gel Merck 60 (70–230 mesh) was used for preparative column chromatography and TLC aluminum foil 60F_254_ for analytical TLC.

### 3.2. Chemistry

*4-Methoxycarbonyl-3-methylisoquinoline-5,8-quinone* (**1**). A suspension of 2,5-dihydroxybenzaldehyde (1 mmol), methyl 3-aminocrotonate (1 mmol), Ag_2_O (2 mmol) and MgSO_4_ (0.5 g) in CH_2_Cl_2_ (25 mL) was stirred at rt for 3 h. The mixture was filtered, the solids were washed with CH_2_Cl_2_ and the solvent removed under reduced pressure. The residue was column cromatographed over silica gel (90:10 CH_2_Cl_2_/AcOEt) to yield pure quinone **1** (86%) as yellow solid, mp 109–111.5 °C; IR ν_max_ 1731 (C=O ester), 1668 and 1575 (C=O quinone); ^1^H-NMR (400 MHz, CDCl_3_): δ 2.65 (s, 3H, Me), 4.03 (s, 3H, CO_2_Me), 7.04 (s, 2H, 6- and 7-H), 9.22 (s, 1H, 1-H); ^13^C-NMR (100 MHz): δ 22.7, 53.1, 122.2, 125.2, 133.4, 138.4, 138.6, 148.6, 161.8, 167.7, 183.38, 183.6; HRMS (M^+^): *m/z* calcd for C_12_H_9_NO_4_: 231.05315; found: 231.05229.

### 3.3. General Procedure for the Synthesis of 6 and 7-Aminoisoquinolinequinone Derivatives

A suspension of isoquinolinequinone **1** (1 mmol), the required amine (2 mmol), CeCl_3_.7H_2_O (0.05 mmol) and ethanol (25 mL) was left with stirring at rt after completion of the reaction as indicated by TLC. The solvent was removed under reduced pressure and the residue was column cromatographed over silica gel (85:15 CH_2_Cl_2_/AcOEt) to yield the corresponding the mixture of regioisomers. These were analysed by ^1^H-NMR to evaluate the proportion between the 6- and 7-aminoisoquinolinequinone derivatives. Column chromatography of the mixture, over silica gel (95:5 CH_2_Cl_2_/ AcOEt), provided pure samples of the regioisomers.

*6- and 7-Phenylamino-4-methoxycarbonyl-3-methylisoquinoline-5,8-quinone* (**2a**,**b**). The mixture of regioisomers was prepared from **1** and aniline (2 h, 74%); red solid; isomer proportion **2a**:**2b** = 66:34.

*Compound* **2a** (less polar, 47%): red solid, mp 197.4–199.3 °C; IR ν_max_ 3449 (N-H), 1718 (C=O ester), 1680 and 1633 (C=O quinone); ^1^H-NMR (400 MHz, CDCl_3_): δ 2.65 (s, 3H, Me), 4.03 (s, 3H, CO_2_Me), 6.39 (s, 1H, 6-H), 7.25 (m, 3H, arom), 7.43 (m, 2H, arom), 7.70 (br s, 1H, NH), 9.24 (s, 1H, 1-H); ^13^C-NMR (100 MHz): δ 23.1, 53.0, 103.7, 121.9, 123.1 (2C), 126.2, 126.6, 130.0(2C), 135.8, 136.8, 144.9, 148.4, 163.3, 168.7, 180.9, 181.5; HRMS (M^+^): *m/z* calcd for C_18_H_14_N_2_O_4_: 322.09534; found: 322.09529.

*Compound* **2b** (23%): red solid, mp 171.0–172.5 °C; IR ν_max_ 3442 (N-H), 1729 (C=O ester), 1692 and 1626 (C=O quinone); ^1^H-NMR (400 MHz, CDCl_3_): δ 2.67 (s, 3H, Me), 4.09 (s, 3H, CO_2_Me), 6.38 (s, 1H, 7-H), 7.25 (m, 3H, arom), 7.42 (m, 2H, arom), 7.45 (br s, 1H, NH), 9.29 (s, 1H, 1-H); ^13^C-NMR (100 MHz): δ 22.8, 53.4, 103.7, 122.8, 123.2 (2C), 125.2, 126.6, 130.1 (2C), 132.4, 137.0, 144.9, 148.8, 160.6, 168.4, 181.7, 182.6; HRMS (M^+^): *m/z* calcd for C_18_H_14_N_2_O_4_: 322.09534; found: 322.09485.

*6- and 7-(4-Methoxyphenyl)amino-4-methoxycarbonyl-3-methylisoquinoline-5,8-quinone* (**3a**,**b**). The mixture of regioisomers was prepared from **1** and *p*-anisidine (1.3 h, 70%); purple solid; isomer proportion **3a**:**3b** = 60:40.

*Compound* **3a** (less polar, 36%): purple solid, mp 164.0–165.5 °C; IR ν_max_ 3349 (N-H), 1738 (C=O ester), 1631(C=O quinone); ^1^H-NMR (400 MHz, CDCl_3_): δ 2.66 (s, 3H, Me), 3.83 (s, 3H, OMe), 4.02 (s, 3H, CO_2_Me), 6.21 (s, 1H, 6-H), 6.94 (d, 2H, *J* = 8.9 Hz, 3'- and 5'-H), 7.18 (d, 2H, *J* = 8.9 Hz, 2'- and 6'-H), 7.58 (br s, 1H, NH), 9.23 (s, 1H, 1-H); ^13^C-NMR (100 MHz): δ 21.1, 53.6, 55.6, 102.8, 115.1 (2C), 122.0, 125.1, 127.0, 129.3, 136.0, 145.7, 148.2, 158.2, 163.0, 168.7, 171.2, 180.9, 181.1; HRMS (M^+^): *m/z* calcd for C_19_H_16_N_2_O_5_: 352.10593; found: 352.10505.

*Compound* **3b** (22%): purple solid, mp 162.5–163.4 °C; IR ν_max_ 3440 (N-H), 1731 (C=O ester), 1613 (C=O quinone); ^1^H-NMR (400 MHz, CDCl_3_): δ 2.67 (s, 3H, Me), 3.83 (s, 3H, OMe), 4.07 (s, 3H, CO_2_Me), 6.20 (s, 1H, 7-H), 6.94 (d, 2H, *J* = 8.8 Hz, 3'- and 5'-H), 7.18 (d, 2H, *J* =8.8 Hz, 2'- and 6'-H), 7.32 (br s, 1H, NH), 9.28 (s, 1H, 1-H); ^13^C-NMR (100 MHz): δ 22.7, 53.7, 55.7, 102.8, 115.2 (2C), 122.9, 125.0, 125.2, 129.3, 129.5, 132.4, 145.7, 148.7, 158.2, 160.3, 171.3, 181.7, 182.3; HRMS (M^+^): *m/z* calcd for C_19_H_16_N_2_O_5_: 352.10593; found: 352.10449.

*6- and 7-(4-Hydroxyphenyl)amino-4-methoxycarbonyl-3-methylisoquinoline-5,8-quinone* (**4a**,**b**). The mixture of regioisomers was prepared from **1** and 4-aminophenol (1.3 h, 75%); purple solid; isomers proportion **4a**:**4b** = 65:35.

*Compound* **4a** (less polar, 42%): purple solid, mp 195.6–197.4 °C; IR ν_max_ 3331 (N-H), 1677 (C=O ester), 1629 (C=O quinone); ^1^H-NMR (400 MHz, CDCl_3_+DMSO): δ 2.77 (s, 3H, Me), 3.93 (s, 3H, CO_2_Me), 6.07 (s, 1H, 6-H), 6.81 (d, 2H, *J* = 8.8 Hz, 2'- and 6'-H), 7.02 (d, 2H, *J* = 8.8 Hz, 3'- and 5'-H), 8.18 (br s, 1H, NH), 9.12 (s, 1H, OH), 9.14 (s, 1H, 1-H); ^13^C-NMR (100 MHz): δ 22.6, 52.8, 102.1, 116.27 (2C), 121.9, 125.2 (2C), 125.7, 127.8, 135.9, 146.1, 147.7, 156.0, 162.3, 168.5, 180.5, 180.7; HRMS (M^+^): *m/z* calcd for C_18_H_14_N_2_O_5_: 338.09027; found: 338.08967.

*Compound* **4b** (19%): purple solid, mp 198.2–199.8 °C; IR ν_max_ 3334 (N-H), 1670 (C=O ester), 1625 (C=O quinone); ^1^H-NMR (400 MHz, CDCl_3_+DMSO): δ 2.59 (s, 3H, Me), 4.05 (s, 3H, CO_2_Me), 6.10 (s, 1H, 7-H), 6.82 (d, 2H, *J* = 8.8 Hz, 2'- and 6'-H), 7.01 (d, 2H, *J* = 8.8 Hz, 3'- and 5'-H); 7.91 (br s, 1H, NH), 9.02 (s, 1H, OH), 9.18 (s, 1H, 1-H); ^13^C-NMR (100 MHz): δ 22.9, 53.0, 102.4, 116.5, 122.0, 125.3 (2C), 125.9, 128.0 (2C), 136.0, 146.1, 148.4, 156.2, 162.7, 168.7, 180.8, 180.9; HRMS (M^+^): *m/z* calcd for C_18_H_14_N_2_O_5_: 338.09027; found: 338.08972.

*7-(4-Acetyloxyphenyl)amino-4-methoxycarbonyl-3-methylisoquinoline-5,8-quinone* (**7a**). A solution of **4a** (1 mmol), acetic anhydride (1.5 mmol) and pyridine (5 mL) was stirred at rt for 2.3 h. The mixture was diluted with H_2_O and extracted with EtOAc. The organic phase was washed with saturated aqueous solution of KHSO_4_, dried (MgSO_4_) and the solvent was removed under reduced pressure. The residue was column cromatographed over silica gel (90:10 CH_2_Cl_2_/AcOEt) to yield **7a** (92%) as a red solid, mp 186.2–187.5 °C; IR ν_max_ 3222 (N-H), 1746 (C=O ester), 1683 and 1625 (C=O); ^1^H-NMR (400 MHz, CDCl_3_): δ 2.32 (s, 3H, OCOMe), 2.66 (s, 3H, Me), 4.02 (s, 3H, CO_2_Me), 6.34 (s, 1H, 6-H), 7.16 (d, 2H, *J* = 8.8 Hz, 2'- and 6'-H), 7.28 (d, 2H, *J* = 8.8 Hz, 3'- and 5'-H), 7.70 (br s, 1H, NH), 9.23 (s, 1H, 1-H); ^13^C-NMR (100 MHz): δ 21.2, 22.7, 53.4, 103.6, 122.6, 123.2 (2C), 124.2 (2C), 125.1, 132.2, 134.4, 144.8, 148.6, 148.7, 160.6, 168.3, 169.4, 181.5, 182.4; HRMS (M^+^): *m/z* calcd for C_20_H_16_N_2_O_6_: 380.10084; found: 380.10043.

*6-(4-Acetyloxyphenyl)amino-4-methoxycarbonyl-3-methylisoquinoline-5,8-quinone* (**7b**). According to the procedure for the preparation of compound **7a**, quinone **7b** was synthesized in 89% yield from **4b**. Compound **7b** was isolated as a pure red solid, mp 144.1–145.8 °C; IR ν_max_ 3309 (N-H), 1732 (C=O ester), 1681 and 1631 (C=O quinone); ^1^H-NMR (400 MHz, CDCl_3_): δ 2.32 (s, 3H, OCOMe), 2.67 (s, 3H, Me), 4.07 (s, 3H, CO_2_Me), 6.34 (s, 1H, 7-H), 7.17 (d, 2H, *J* = 8.8 Hz, 2'- and 6'-H), 7.27 (d, 2H, *J* = 8.8 Hz, 3'- and 5'-H), 7.41 (br s, 1H, NH), 9.29 (s, 1H, 1-H); ^13^C-NMR (100 MHz): δ 21.1, 23.0, 53.2, 103.6, 121.8, 123.1 (2C), 124.2 (2C), 126.0, 134.2, 135.6, 144.9, 148.3, 148.6, 163.2, 168.6, 169.4, 180.7, 181.3; HRMS (M^+^): *m/z* calcd for C_20_H_16_N_2_O_6_: 380.10084; found: 380.10015.

### 3.4. General Procedure for the Synthesis of 6- and 7-Bromo Derivatives

A solution of 6- or 7-phenylaminoisoquinolinequinone (1 mmol), the corresponding *N*-bromosuccinimide (NBS) (1 mmol) and methanol (15 mL) was left with stirring at rt after completion of the reaction as indicated by TLC. The solvent was removed under reduced pressure and the residue was column cromatographed over silica gel (90:10 CH_2_Cl_2_/AcOEt) to yield the corresponding haloisoquinolinequinone derivative.

*6-Bromo-7-phenylamino-4-methoxycarbonyl-3-methylisoquinoline-5,8-quinone* (**5a**). Prepared from **2a**and NBS (15 min, 87%): dark red solid, mp 188.1–190.5 °C; IR ν_max_ 3311 (N-H), 2955 and 2926 (C-H), 1732 (C=O ester), 1683 and 1641 (C=O quinone); ^1^H-NMR (400 MHz, CDCl_3_): δ 2.68 (s, 3H, Me), 4.06 (s, 3H, CO_2_Me), 7.05 (m, 2H, arom), 7.33 (m, 3H, arom), 7.88 (s, 1H, NH), 9.25 (s, 1H, 1-H); ^13^C-NMR (100 MHz): δ 23.2, 53.6, 106.6, 121.2, 125.4 (2C), 126.8, 128.8 (2C), 131.9, 134.6, 136.8, 144.5, 148.8, 163.5, 168.2, 175.6, 178.8; HRMS (M^+^): *m/z* calcd for C_18_H_13_N_2_O_4_Br: 400.00586; found: 400.00553.

*7-Bromo-6-phenylamino-4-methoxycarbonyl-3-methylisoquinoline-5,8-quinone* (**5b**). Prepared from **2b** and NBS (15 min, 74%): purple solid, mp 175.4–176.6 °C; IR ν_max_ 3263 (N-H), 2953 and 2922 (C-H), 1734 (C=O ester), 1686 and 1633 (C=O quinone); ^1^H-NMR (400 MHz, CDCl_3_): δ 2.67 (s, 3H, Me), 4.01 (s, 3H, CO_2_Me), 7.01 (m, 2H, arom), 7.33 (m, 3H, arom), 7.62 (s, 1H, NH), 9.33 (s, 1H, 1-H); ^13^C-NMR (100 MHz): δ 22.8, 53.4, 108.3, 122.2, 124.8 (2C), 126.3, 128.8 (2C), 131.8, 132.4, 137.1, 144.4, 149.3, 161.2, 167.8, 176.0, 179.4; HRMS (M^+^): *m/z* calcd for C_18_H_13_N_2_O_4_Br: 400.00586; found: 400.00585.

*6-Bromo-7-(4-methoxyphenyl)amino-4-methoxycarbonyl-3-methylisoquinoline-5,8-quinone* (**6a**). Prepared from **3a** and NBS (30 min, 80%): purple solid, mp 160.2–162.5 °C; IR ν_max_ 3263 (N-H), 2956 and 2836 (C-H), 1724 (C=O ester), 1684 and 1637 (C=O quinone); ^1^H-NMR (400 MHz, CDCl_3_): δ 2.67 (s, 3H, Me), 3.83 (s, 3H, OMe), 4.06 (s, 3H, CO_2_Me), 6.88 (d, 2H, *J* = 8.8 Hz, 3'- and 5'-H), 7.06 (d, 2H, *J* = 8.8 Hz, 2'- and 6'-H), 7.88 (br s, 1H, NH), 9.23 (s, 1H, 1-H); ^13^C-NMR (100 MHz): δ 23.2, 53.4, 55.6, 105.1, 113.9 (2C), 121.2, 126.8, 127.2 (2C), 129.5, 134.8, 144.3, 148.8, 158.6, 163.5, 168.3, 175.4, 178.8; HRMS (M^+^): *m/z* calcd for C_19_H_15_N_2_O_5_Br: 430.01642; found: 440.01596.

*7-Bromo-6-(4-methoxyphenyl)amino-4-methoxycarbonyl-3-methylisoquinoline-5,8-quinone* (**6b**). Prepared from **3b** and NBS (30 min, 78%): purple solid, mp 163.3–164.5 °C; IR ν_max_ 3238 (N-H); 2923 and 2854 (C-H), 1729 (C=O ester); 1685 and 1641 (C=O quinone); ^1^H-NMR (400 MHz, CDCl_3_): δ 2.70 (s, 3H, Me), 3.86 (s, 3H, OMe), 4.05 (s, 3H, CO_2_Me), 6.87 (d, 2H, *J* = 8.8 Hz, 3'- and 5'-H), 7.03 (d, 2H, *J* = 8.8 Hz, 2'- and 6'-H), 7.58 (br s, 1H, NH), 9.35 (s, 1H, 1-H); ^13^C-NMR (100 MHz): δ 22.8, 53.5, 55.6, 106.5, 114.0 (2C), 122.3, 125.4, 126.9 (2C), 129.8, 132.2, 144.4, 149.4, 158.4, 161.1, 168.0, 176.0, 179.6; HRMS (M^+^): *m/z* calcd for C_19_H_15_N_2_O_5_Br: 430.01642; found: 430.01517.

*6-Bromo-7-(4-acetyloxyphenyl)amino-4-methoxycarbonyl-3-methylisoquinoline-5,8-quinone* (**8**). Prepared from **7a** and NBS (20 min, 56%): red solid, mp 179.5–180.8 °C; IR ν_max_ 3282 (N-H), 1753 (C=O ester), 1731 (C=O acetyl), 1679 and 1629 (C=O quinone); ^1^H-NMR (400 MHz, CDCl_3_): δ 2.32 (s, 3H, OCOMe), 2.68 (s, 3H, Me), 4.06 (s, 3H, CO_2_Me), 7.12 (s, 4H, arom), 7.84 (br s, 1H, NH), 9.25 (s, 1H, 1-H); ^13^C-NMR (100 MHz): δ 21.3, 23.2, 53.5, 106.6, 121.2, 121.9 (2C), 126.4 (2C), 126.8, 134.2, 134.6, 144.1, 148.9, 149.1, 163.6, 168.2, 169.3, 175.6, 178.7; HRMS (M^+^): *m/z* calcd for C_20_H_15_N_2_O_6_Br: 458.01135; found: 458.01006.

*6-Bromo-7-(4-hydroxyphenyl)amino-4-methoxycarbonyl-3-methylisoquinoline-5,8-quinone* (**9**). A suspension of **8**, K_2_CO_3_ (15 mg) in MeOH (5 mL) was stirred at rt for 1 h, the reaction mixture was partitioned between EtOAc/water and the organic layer was washed with water (3 × 15 mL). The dried extract was evaporated under reduced pressure and the residue was column cromatographed over silica gel (90:10 CH_2_Cl_2_/AcOEt) to give compound **9** (64%) as purple solid, mp 190.5–192.1 °C; IR ν_max_ 3328 (N-H), 1753 (C=O ester), 1618 (C=O quinone); ^1^H-NMR (400 MHz, (CD_3_)_2_CO): δ 2.58 (s, 3H, Me), 3.96 (s, 3H, CO_2_Me), 6.83 (d, 2H, *J* = 8.7 Hz, 3'- and 5'-H), 7.11 (d, 2H, *J* = 8.7 Hz, 2'- and 6'-H), 8.52 (s, 1H, OH), 8.79 (br s, 1H, NH), 9.13 (s, 1H, 1-H); ^13^C-NMR (100 MHz): δ 22.9, 53.2, 105.1, 115.8 (2C), 123.0, 127.1, 128.2 (2C), 130.8, 135.6, 146.6, 149.0, 156.9, 162.9, 168.8, 175.7, 179.8; HRMS (M^+^): *m/z* calcd for C_18_H_13_N_2_O_5_Br: 416.00078; found: 415.99527.

### 3.5. Antiproliferative Assay

The cell lines used in this work were obtained from the American Type Culture Collection (ATCC. Manasas, VA, USA). They included MRC-5 normal human lung fibroblasts (CCL-171), AGS human gastric adenocarcinoma cells (CRL-1739), SK-MES-1 human lung cancer cells (HTB-58) and J82 human bladder carcinoma cells (HTB-1). After the arrival of the cells, they were proliferated in the corresponding culture medium as suggested by the ATCC. The cells were stored in medium containing 10% glycerol in liquid nitrogen. The viability of the cells after thawing was higher than 90%, as assessed by trypan blue exclusion test. Cells were sub-cultured once a week and the medium was changed every two days. Cells were grown in the following media: MRC-5, SKMES-1, and J82 in Eagle’s minimal essential medium (EMEM) and AGS cells in Ham F-12. The EMEM medium contained 2 mM L-glutamine, 1 mM sodium pyruvate and 1.5 g/L sodium hydrogen carbonate. Ham F-12 was supplemented with 2 mM L-glutamine and 1.5 g/L sodium hydrogen carbonate. All media were supplemented with 10% heat-inactivated FBS, 100 IU/mL penicillin and 100 μg/mL streptomycin in a humidified incubator with 5% CO_2_ in air at 37°C. For the experiments, cells were plated at a density of 50,000 cells/mL in 96-well plates. One day after seeding, the cells were treated with the medium containing the compounds at concentrations ranging from 0 up to 100 μM during 3 days. The concentrations used to calculate the IC_50_ values were: 100, 50, 25, 12.5, 6.25, 3.125, 1.56, 0.78, 0.39, 0.195 and 0.00 µM. The compounds were dissolved in DMSO (1% final concentration) and complete medium. Untreated cells (medium containing 1% DMSO) were used as controls. At the end of the incubation, the MTT reduction (3-(4,5-dimethylthiazol-2-yl)-2,5-diphenyltetrazolium bromide) assay was carried out to determine cell viability. The final concentration of MTT was 1 mg/mL. MTT metabolite was dissolved adding 100 µL of ethanol (acidified with HCl). The plates were shaken for 10 min and the absorbance was measured at 550 nm using a Universal Microplate Reader (EL_X_ 800, Bio-Tek Instruments Inc., Winnoski, VT, USA). Six replicates for each concentration were used and the values were averaged. The results were transformed to percentage of controls and the IC_50_ values were graphically obtained from the dose-response curves. The IC_50_ value was obtained adjusting the dose-response curve to a sigmoidal model (a + (b – a)/1 + 10^(x−c)^), where c = log IC_50_.

## 4. Conclusions

We have reported the synthesis and biological evaluation as antiproliferative agents of a broad variety of isoquinoline-5,8-quinones substituted at the quinone nucleus with phenylamino and bromine groups. The new substituted isoquinolinequinones were prepared through efficient amination and bromination substitution reactions from 4-methoxycarbonyl-3-methylisoquinoline-5,8-quinone (**1**). The members of this series expressed moderate to high *in vitro* antiproliferative activity against three human cancer cell lines: AGS (gastric), SK-MES-1 (lung) and J82 (bladder) cell lines. From the current investigation, structure-activity relationships of the phenylaminoisoquinolinequinone series demonstrate that a phenylamino group substituted at the quinone nucleus of the isoquinolinequinone pharmacophore promotes a high increase of the antiproliferative activity on gastric, lung and bladder cancer cell lines. The substitution effects are more significant in enhancing the antiproliferative activity for those members containing the phenylamino group at C-6. The insertion of a bromine atom at the quinone nucleus of the phenylaminoquinones decreases the antiproliferative activity compared to that of their precursors, except for aminoquinone **2a**.

The large antiproliferative potencies of the 6-substituted regioisomers **2b**, **3b**, **4b** and **7b**, with higher half-wave potentials (less negative E^I^_1/2_), compared to the 7-substituted derivatives **2a**, **3a**, **4a** and **7a** on gastric and lung cancer cell lines suggest that their biological activity involves a redox cycling process. Compounds **3b**, **4b**, **7b** and **5a** are the most significant antiproliferative active members of the series. Among these substances, the member **4b** is the most potent congener by its submicromolar IC_50_ values against the three tested cell lines.
